# Supplementary material for: Italian Ryegrass as a Forage Crop for the Baltics: Opportunities and Challenges in Light of Climate Change
Source: Plants (Basel). 2023 Nov 13;12(22):3841. doi: 10.3390/plants12223841 (PMC10674386; doi:10.3390/plants12223841)
Supplement: Supplementary file 1 [file plants-12-03841-s001.zip › plants-2674607-supplementary.pdf]

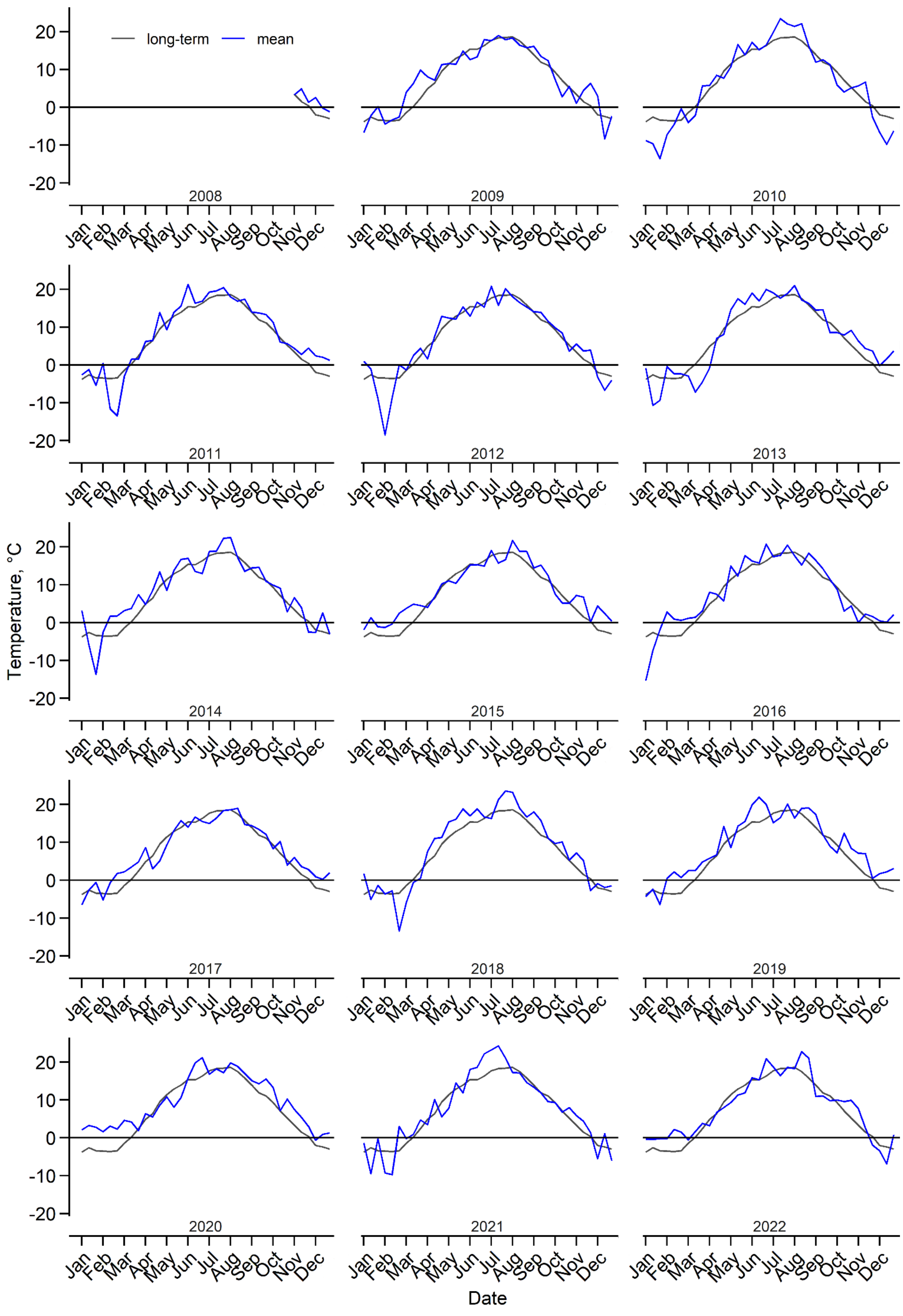

Figure S1. Mean air temperatures over the period 2008 November – 2022 December at the experimental site (Dotnuva meteorological station, Lithuania).

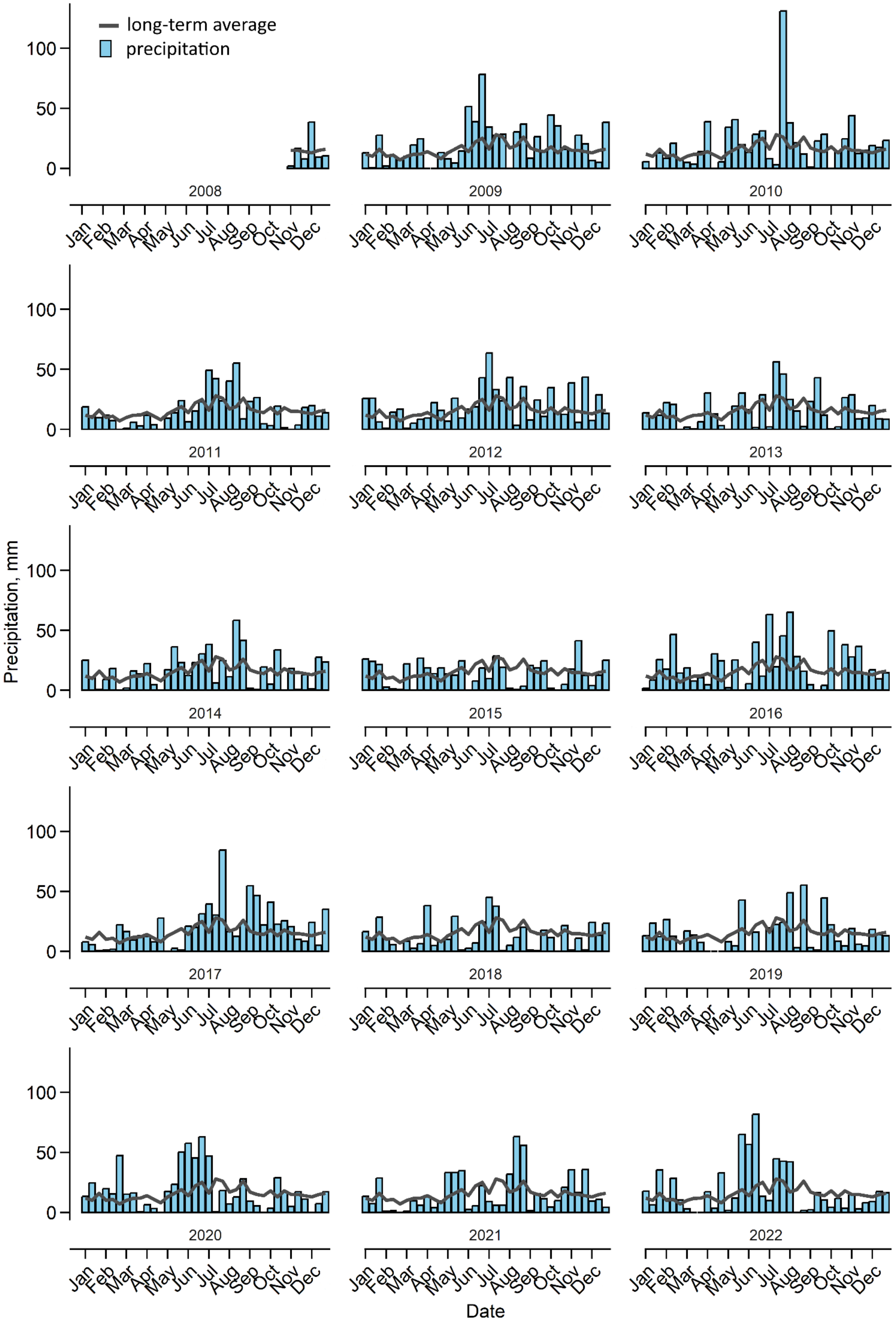

Figure S2. Precipitation over the period 2008 November – 2022 December at the experimental site (Dotnuva meteorological station, Lithuania).

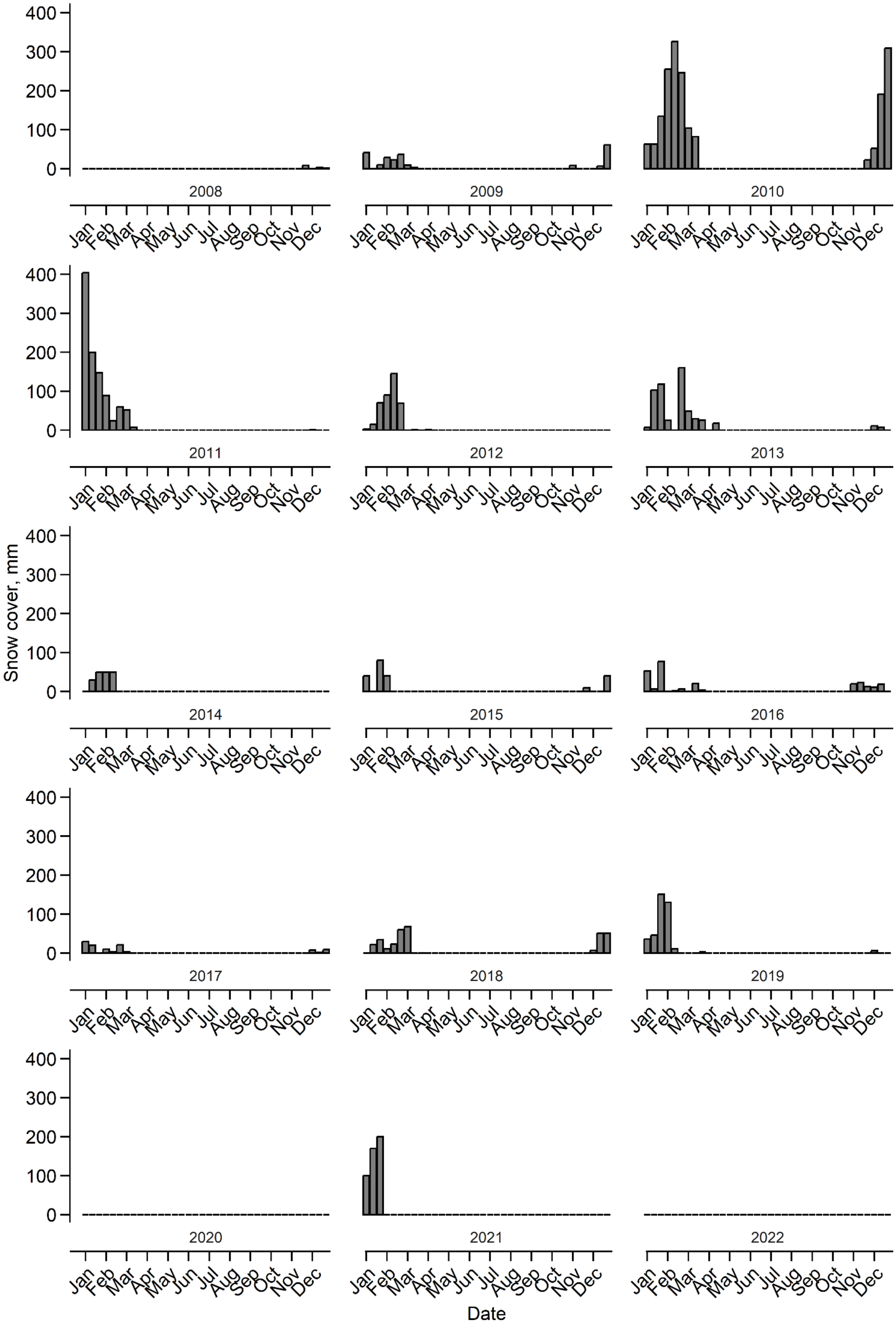

Figure S3. Snow cover over the period 2008 November – 2022 December at the experimental site (Dotnuva meteorological station, Lithuania).

Table S1. The main agrochemical soil characteristics before the sowing at the experimental site, 2008–2021.

Soil characteristic/ year	2008	2009	2010	2011	2012	2013	2015	2016	2017	2018	2019	2020	2021
P_2O_5, kg ha^{-1}	220	220	250	270	206	206	207	206	210	219	206	206	215
K_2O, kg ha^{-1}	101	136	140	108	154	126	150	150	149	153	154	150	153
pH	7.3	7.3	7.2	7.3	7	7.3	7.3	7.3	7.3	7.3	7.3	7.3	7.3
Humus, %	2.1	1.9	2.2	2.2	2.1	2.2	1.9	2.2	2.2	2.2	2.1	2.1	2.2
